# Values, preferences and current hepatitis B and C testing practices in low- and middle-income countries: results of a survey of end users and implementers

**DOI:** 10.1186/s12879-017-2769-y

**Published:** 2017-11-01

**Authors:** Elena Ivanova Reipold, Alessandra Trianni, Douglas Krakower, Stefano Ongarello, Teri Roberts, Philippa Easterbrook, Claudia Denkinger

**Affiliations:** 1 FIND, Geneva, Switzerland; 2 Beth Israel Deaconess Medical Center, Harvard Medical School, Boston, MA USA; 3 World Health Organization, Geneva, Switzerland; 4 MSF Access Campaign, Geneva, Switzerland

## Abstract

**Background:**

Access to hepatitis B virus (HBV) and hepatitis C virus (HCV) diagnostics remains a key bottleneck in scale-up of access to HBV and HCV treatment, particularly in low- and middle-income countries (LMICs) that lack laboratory resources and skilled personnel. To inform the development of World Health Organization (WHO) testing guidelines on who to test and how to test, we performed a “values and preferences” survey of end users and implementers of hepatitis testing in LMICs on current hepatitis B and C testing practices and acceptability of diagnostic approaches, as well as preferences for the future.

**Methods:**

The survey consisted of a four-part, 28 question online survey tool using SurveyMonkey software. The invitation to participate was sent via email to a network of contacts in hepatitis clinical care, research, advocacy and industry.

**Results:**

The survey collected responses on current testing practices from 48 respondents in 23 LMICs. Only a small proportion of hepatitis testing is currently funded through government-supported programmes. Most limit their testing programmes to blood donor screening and although testing is recommended in several populations, this is not well implemented. Also, there is still very limited access to virological testing.

**Conclusions:**

The survey showed that HBV and HCV testing programmes in LMICs are inadequate and/or scarce. Lack of affordable diagnostic tests; lack of funding, public education and awareness; absence of national policies and guidelines; and a dearth of skilled health professionals are the most important barriers to scaling up HBV and HCV diagnosis and treatment.

**Electronic supplementary material:**

The online version of this article (10.1186/s12879-017-2769-y) contains supplementary material, which is available to authorized users.

## Background

Hepatitis B (HBV) and hepatitis C (HCV) virus infections are major causes of chronic liver disease worldwide, resulting in an estimated 1.34 million deaths per year [1]. According to current estimates, approximately 257 million people globally are chronically infected with HBV (defined as persistence of HBV surface antigen (HBsAg) for more than 6 months) and 71 million have chronic HCV infection [1]. Over 80% of the people affected are living in low- and middle-income countries (LMICs) [1]. HCV treatment has been transformed by the availability of new all-oral, highly potent regimens using direct antiviral agents (DAA) with high rates of cure. Effective therapy with tenofovir or entecavir for HBV is also available but requires lifelong treatment and monitoring. Both HBV and HCV treatments are becoming increasingly available in LMICs, but a major barrier to accessing treatment and care is very limited access to hepatitis testing, meaning that many people who are infected are unaware of their status [2, 3]. In addition, existing diagnostic algorithms are complex and virological tests costly.

The standard diagnostic algorithm to determine chronic HBV infection involves HBsAg testing in serum or plasma using laboratory-based chemiluminescence enzyme immunoassay (CLIA) or rapid diagnostic tests (RDT) in settings where laboratory infrastructure and trained personnel are inadequate. HBV DNA measurement is also needed together with liver functions to stage liver disease and indicate treatment [4].

The current approach for diagnosis of HCV infection consists of initial screening for evidence of past or current HCV infection with a serological assay (either a CLIA or an RDT) that detects hepatitis C antibody. [5–7]. Those with positive serology will need further testing to confirm the presence of HCV viraemia, since 15–30% of HCV-infected individuals spontaneously clear the virus following infection, remaining HCV antibody positive without being viraemic [8]. To identify those with chronic HCV, confirmatory testing for HCV RNA or HCV core antigen is performed. Most of this testing is performed in highly resourced settings and is not accessible to the majority of HCV-infected individuals in LMICs.

There is an urgent need for evidence-based guidance for simplified and more affordable HBV and HCV testing in various LMIC settings, including centralized laboratories and point-of-care settings such as health centres and mobile clinics.

Development of the 2017 WHO Guidelines on hepatitis B and C testing [9] and formulation of recommendations were based on the GRADE system (Grading of Recommendations, Assessment, Development and Evaluation) [10]. Key domains considered included the nature and quality of evidence, balance of benefits and harms, acceptability or values and preferences of end users, resource use and programmatic feasibility. To provide data on end-user perspectives and acceptability, we performed a “values and preferences” survey of end users and implementers of hepatitis testing in LMICs on current hepatitis B and C testing practices and diagnostic approaches, and preferences for the future.

## Methods

### Questionnaire development

To assess the values and preferences for different testing strategies and approaches, a four-part 28 question online survey tool was developed by FIND and WHO with input from Médecins Sans Frontières (MSF), Public Health England and Médecins du Monde. Part One (four questions) collected information on professional profiles and experience in viral hepatitis testing of survey respondents; Part Two (eight questions) focused on current hepatitis B and C testing practices in the respondent’s geographic area; Part Three (12 questions) and Part Four (four questions) collected responses on preferences for future hepatitis testing practices and for test of cure, respectively. For questions in Part Two and Three, only responses related to testing practices in LMICs were collected. Survey questions were designed as multiple-choice options with opportunity for comments. The survey was developed online using Survey Monkey software (SurveyMonkey Inc., Palo Alto, USA) (see Additional file 1).

### Survey administration

Invitation to participate was sent via email to 306 people on the WHO Hepatitis database including clinicians, patient organizations, civil society representatives, programme managers, policy-makers and pharmaceutical industry employees. The web link to the survey was also provided via FIND and HIV Forum (USA) newsletters. Respondents had 3 weeks to answer the questions. The responses were exported into a Microsoft Excel table (Microsoft Office 2010, Microsoft Corporation, Redmond, WA, USA) and statistical analysis was performed using R software (version 3.2, R Foundation for Statistical Computing, Vienna, Austria).

Information on preferences for future hepatitis testing collected in Part Three and Four of the survey was analysed by calculating the percentage of respondents who selected each response choice. In contrast, data on current hepatitis testing practices collected in Part Two was analysed by calculating the percentage of countries represented in the responses. When two or more respondents from the same country participated in the survey, only responses from participants who indicated that they are familiar with testing practices at a national level were taken into the final analysis. The responses were merged and in cases where answers did not match, the “yes” (or “available”) rather than “no” (or “not available”) answers were included in the analysis, since it was assumed that “no” or “not available” answers could be due to incomplete knowledge about diagnostic practices on a national level.

Some of the findings around user preferences for future hepatitis C diagnostics were used to complement recently developed target product profiles for HCV diagnostic solutions that can be performed at or near the site of patient care (point-of-care testing, also referred to as near-patient testing) and are published in a separate report within this Supplement [11].

## Results

### Characteristics of respondents

There were 104 respondents from 43 countries (Table 1). Forty-eight respondents represented 23 LMICs (20 respondents provided answers for nine upper-middle income, 24 respondents for 10 lower-middle income and four respondents for four low-income countries). Nine of the countries were in Asia or Asian Pacific, six in sub-Saharan Africa, one I North Africa, four in Eastern Europe, two in South America and one in North Africa.

**Table 1:** Countries who participated in the survey

High-income	Upper middle-income	Lower middle-income	Low-income
Argentina	Brazil	Burma	Cambodia
Australia	Bulgaria	Egypt	Mali
Austria	China	Georgia	Uganda
Bahrain	Macedonia	India	Zimbabwe
Canada	Malaysia	Indonesia	
Germany	Peru	Kenya	
Greece	Serbia	Nigeria	
Hong Kong	South Africa	Pakistan	
Italy	Turkey	Papua New Guinea	
Japan		Vietnam	
Korea, South			
Latvia			
Netherlands			
Russia			
Slovenia			
Spain			
Sweden			
Switzerland			
United Kingdom			
United States of America			
Total: 20	Total: 9	Total: 10	Total: 4

The majority (20) were high income countries while 23 were LMIC. Countries were categorized according to World Bank Country Groups [21]

Almost half of respondents were medical doctors (43 out of 104) and 21 of these were working in a research capacity. The remaining 61 participants had a non-medical background and included 18 researchers, eight laboratory experts and 17 civil society activists (Table 2). Twenty two respondents indicated that they were employees of a national or international non-governmental organization, six were working with national government programmes, and six were representatives of the *in vitro* diagnostic industry. Other professional profiles included members of the National Expert Committee for Viral Hepatitis, peer educators with injecting drug user training and employees of pharmaceutical companies. The majority of respondents had more than 10 years’ experience in the viral hepatitis field (Table 2).

**Table 2:** Characteristics of survey respondents

Characteristic	Number of respondents (%)
Professional profile*:	
Medical doctor/clinical officer	43 (41.4%)
Primary care provider	5 (4.8%)
Laboratory expert	8 (7.7%)
Researcher	39 (37.5%)
*In vitro* diagnostics industry personnel	6 (5.8%)
Employee of an international organization (e.g., WHO)	2 (1.9%)
National programme administrator	6 (5.8%)
Employee/Consultant of a national or international NGO	22 (21.2%)
Programme implementer	13 (12.5%)
Policy maker	9 (8.7%)
Civil Society Activist	17 (16.4%)
Other	17 (16.4%)
Expertise in viral hepatitis field:	
Less than 1 year	7 (6.7%)
1–2 years	11 (10.6%)
3–5 years	19 (18.3%)
5–10 years	2 (1.9%)
More than 10 years	65 (62.5%)
Total	104
Information provided about HBV services:	
National level	30 (63.8%)
Province/state	4 (8.5%)
Specific sites/programme	10 (21.3%)
Total	47
Information provided HCV services:	
National level	32 (66.7%)
Province/state	4 (8.3%)
Specific sites/programmes	10 (20.8%)
Total	48

*checking more than one box was allowed and therefore the total number of answer choices exceeds the total number of respondents (*n* = 104)

More than 60% of respondents provided information about HBV (63.8%) and/or HCV (66.7%) testing practices at a national level and 21.3% and 20.8% at specific site/programme level (Table 2). For four out of the 23 LMICs (Cambodia, Mali, Uganda, Zimbabwe), no respondents familiar with HBV and HCV testing practices at national level participated in the survey. For one country (Georgia), responses on HCV but not HBV testing practices at national level were received.

### Existing hepatitis testing practices in countries

#### Provision of testing

For the majority (over 72%) of countries where information was available, HBV and HCV testing is offered by both the public and private health sector (Table 3). A survey participant from Macedonia reported that testing is provided by the public sector only. HBV testing in two countries and HCV testing in three countries was reported to be available only in the private sector. The response from Mali suggested that HCV and HBV testing was only being provided by NGOs. It should be noted that we did not have any respondents from Zimbabwe, Uganda and Mali providing answers on testing practices at national level.

**Table 3:** Viral hepatitis testing services providers and funding

	Number of LMIC (% of LMIC represented in the survey)	
	HBV	HCV
Testing service provider		
Public/government sector and private sector	15 (75%)	16 (72%)
Public sector and NGO	1 (5%)	0
Private sector only	2 (10%)	3 (13%)
Public sector only	1 (5%)	1 (4.5%)
Private and NGO or NGO only	1 (5%)	2 (9%)
Total	20	22
Staff providing hepatitis testing		
Highly skilled staff and less trained health-care workers	10 (50%)	11 (50%)
Highly skilled staff only	9 (45%)	10 (45%)
Other	0	1 (4.5%)
Not sure	1 (5%)	0
Total	20	22
Funding for testing		
Patients (i.e., self-funded or private insurance)	8 (40%)	10 (45%)
Partially by government	12 (60%)	11 (50%)
Other	0	1 (4.5%)
Total	20	22

#### Provision of treatment

Treatment for HCV is provided in all LMICs represented in the survey responses, mainly in national and regional hospitals. In four countries, treatment is reported to be available exclusively in the private sector and in Papua New Guinea and India it is also provided by some NGOs (see Additional file 2).

#### Funding for testing

Although respondents indicated that HBV and HCV testing is partially paid for by the public health services in half of the LMICs represented in the survey (Table 3), in many cases government funding (e.g. India, Indonesia) only covers certain populations. According to the survey responses, in seven out of 23 countries (Bulgaria, Myanmar, Cambodia, China, Uganda, Kenya, Mali), both HBV and HCV testing is funded through patient self-pay. In five countries (India, Papua New Guinea, Serbia, Vietnam and Zimbabwe), HBV testing is at least partially paid for by the public health services, while HCV testing is paid exclusively by patients.

#### Staff providing testing

In half of the LMICs represented in the survey responses, HBV/HCV testing is performed by both highly trained medical personnel (lab technicians, physicians, nurses) and less trained health-care workers. In the other half (e.g. Georgia, Bulgaria, Macedonia, Turkey, Vietnam, Myanmar), testing is done exclusively by skilled medical personnel (Table 3).

#### Populations targeted for testing

**Fig. 1:**
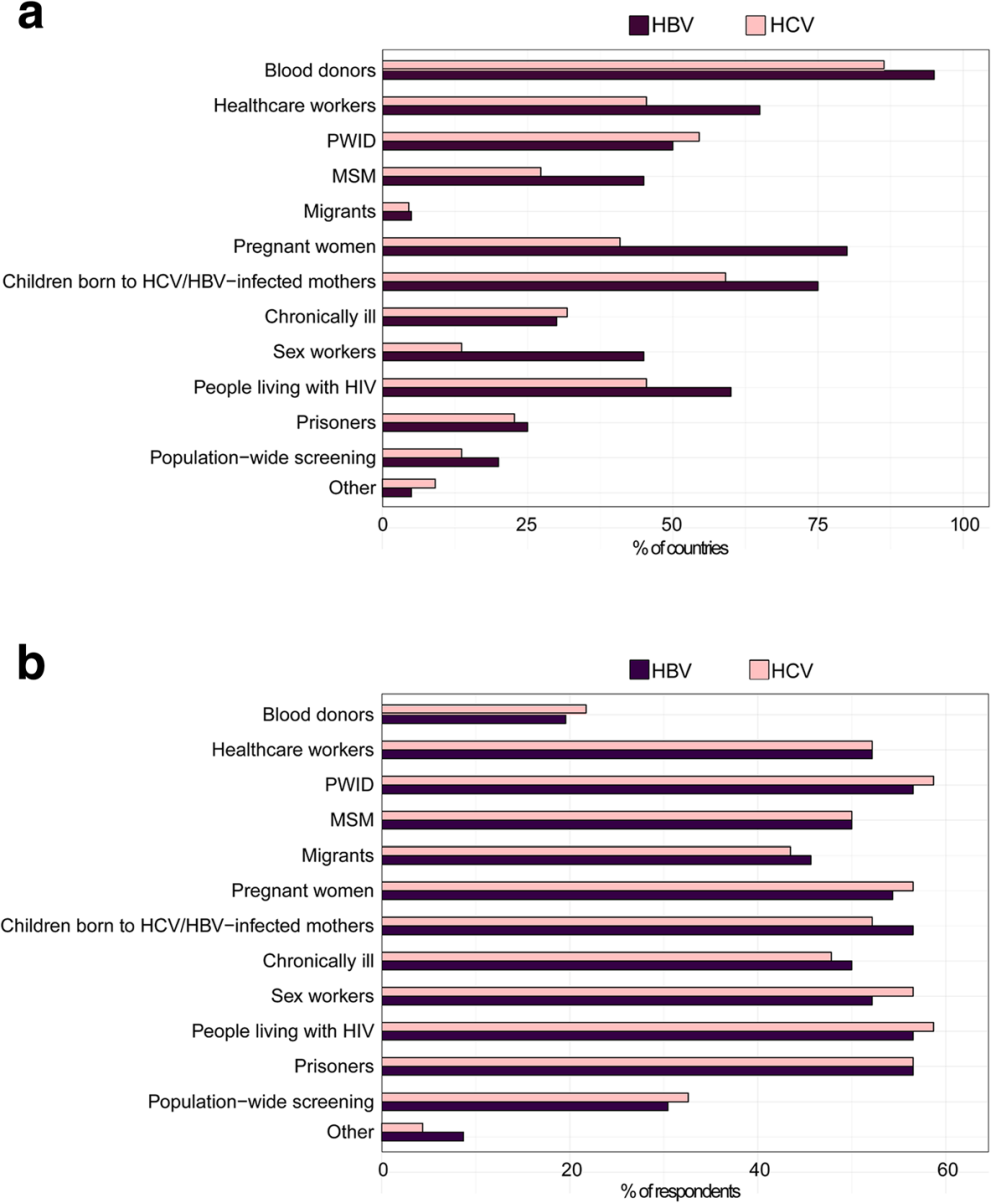
Target populations for hepatitis B and C testing. **a** Population that for which HBV (purple) or HCV (pink) testing is currently established in LMIC represented in the survey responses. Values are given in per cent of LMIC from which responses that testing is established were received (*n* = 23 LMIC). When more than one respondent have provided answers for one country, the responses were merged (see Methods). **b** Populations for which testing programmes need to be established in priority according to survey respondents. Values are given in per cent of respondents (*n* = 48 respondents)

Respondents were asked whether some specific populations are targeted for HBV and HCV testing in their countries. In more than 80% of countries, blood donors are routinely tested for HBV and HCV (Figure 1a). Screening of health-care workers (HBV in 65% of the countries and HCV in 45%), pregnant women (HBV in 80% and HCV in 41%), children born to HBV/HCV-infected mothers (HBV in 75% and HCV in 59%), PWID (HBV in 50% and HCV in 54%) and persons living with HIV (HBV in 60% and HCV in 45%) were also recommended in at least half of the countries, but respondents noted that it is generally doctor-initiated and erratically implemented. Only three countries − Egypt, Nigeria and Turkey − indicated they had a population-based screening programme. Respondents from Zimbabwe and Vietnam indicated that there was no systematic HCV screening for any target population.

When asked which populations in countries where a testing programme is not currently established should be prioritised for testing in the future, more than 50% of respondents considered that testing should be implemented for most of the target populations listed (Figure 1b).

#### Testing assays and algorithms

Survey responses showed that in more than half of the LMICs represented in the survey, a testing algorithm for a positive HBsAg or HCV antibody serological assay, followed by a virological confirmatory assay, is recommended (HBV DNA, 57%, or HCV RNA, 61%) (see Additional file 2). However*,* while testing using a serological assay was available in the majority of testing programmes, access to supplementary HBV DNA and HCV RNA testing was very limited and available in only 5 to 30% of reporting countries, depending on the population (Table 4). HBV DNA was used within certain programmes in seven out of 20 countries (India, Macedonia, Malaysia, South Africa, Turkey, Georgia and Nigeria) and HCV RNA only in five out of 22 countries (Georgia, Indonesia, Malaysia, South Africa and Turkey). Systematic use of HCV core antigen testing was not reported for any of LMICs represented in the survey.

**Table 4:** Types of testing used for different programmes

Targeted population	Number of LMIC (% of LMIC)*						
	HBV testing			HCV testing			
	RDT/EIA/RIA	DNA	FS	RDT/EIA/RIA	RNA	cAg	FS
Blood donors	18 (90%)	5 (25%)	0	19 (86.4%)	5 (22.7%)	0	1 (4.5%)
Health-care workers	12 (60%)	3 (15%)	0	10 (45.5%)	1 (4.5%)	0	0
People who inject drugs	9 (45%)	3 (15%)	0	11 (50%)	1 (4.5%)	0	1 (4.5%)
Men who have sex with men	8 (40%)	1 (5%)	0	6 (27.3%)	0	0	0
Migrants	0	0	0	1 (4.5%)	0	0	0
Pregnant women	15 (75%)	2 (10%)	0	7 (31.8%)	1 (4.5%)	0	0
Children born to HCV/HBV-infected mothers	14 (70%)	4 (20%)	0	12 (54.5%)	3 (13.6%)	0	1 (4.5%)
Chronically ill	6 (30%)	1 (5%)	0	7 (31.8%)	0	0	0
Commercial sex workers	8 (40%)	1 (5%)	0	3 (13.6%)	0	0	0
People living with HIV	12 (60%)	6 (30%)	1 (5%)	9 (40.9%)	1 (4.5%)	0	1 (4.5%)
Prisoners	4 (20%)	1 (5%)	1 (5%)	5 (22.7%)	1 (4.5%)	0	1 (4.5%)
Population-wide testing	4 (20%)	1 (5%)	1 (5%)	2 (9.1%)	0	0	0
Testing is not a part of any programme	1 (5%)	0	0	1 (4.5%)	1 (4.5%)	0	1 (4.5%)
Other	1 (5%)	1 (5%)	1 (5%)	1 (4.5%)	0	0	0
Total	20			22			

*number and per cent of LMIC refer to LMIC represented in the survey responses

RDT/EIA/RIA – rapid diagnostic test/enzyme immunoassay/radioimmunoassay; DNA – HBV DNA test; RNA – HCV RNA test; cAg – HCV core antigen test; FS – Fibrosis staging

Fibrosis staging analysis of liver disease was reported in just two countries − in Georgia for HBV/HIV- and HCV/HIV-coinfected persons, children born to HIV/HBV infected mothers, PWID and blood donors, and in Macedonia for all populations.

#### Test brands

Commonly used test brands for HBsAg and HCV antibody serology across different hepatitis testing programmes included SD Bioline (Standard Diagnostics, South Korea), TRI-DOT (J. Mitra, India), ASSURE (MP Biomedicals, Santa Ana, CA, USA) and Vikia (BioMérieux, France). Abbott Architect (Abbott Laboratories, Abbott Park, IL, USA) platforms are available and used in South Africa for HBV antigen and HCV serology testing and in India, Indonesia, Macedonia, Vietnam, Turkey for HCV core antigen testing.

For HBV DNA and HCV RNA testing, countries were using Roche COBAS (Roche Diagnostics, Indianapolis, IN, USA) and Abbott RealTi*me* (Abbott Laboratories, Abbott Park, IL, USA) platforms. For fibrosis staging analysis, most of the countries have access to FibroScan together with liver biopsy and routine blood tests (e.g. AST/ALT ratio, APRI, FIB-4). Survey responses received from Mali, Pakistan and Uganda indicate that only routine blood tests are available in these countries.

### Preferences for future approaches to testing to optimise access

**Fig. 2:**
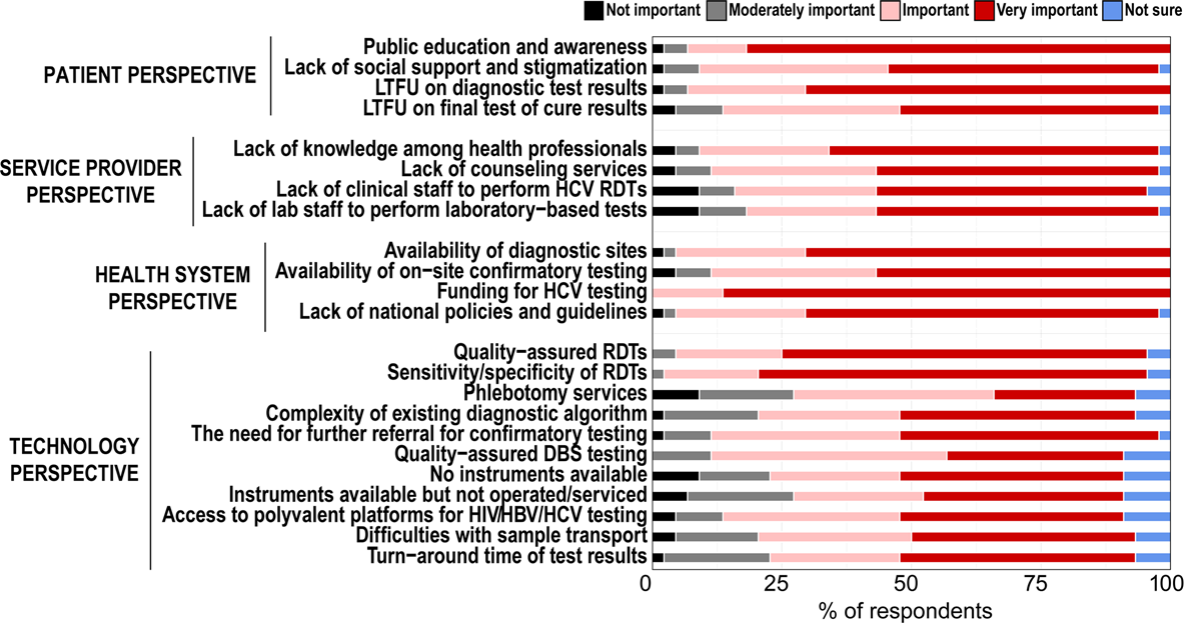
Issues that need to be addressed in order to establish large scale access to HCV diagnostics in LMIC

Respondents were asked to weigh the most critical issues that need to be addressed to improve access to HCV diagnostics in LMICs. The majority of respondents defined lack of public education and awareness, loss to follow-up on diagnostic test results, lack of knowledge among health professionals, limited availability of diagnostic sites, lack of funding for HCV testing and lack of quality-assured RDT for serology testing as very important barriers to large scale access to HCV diagnostics (Figure 2).

Respondents were also asked about the optimal timing of the test of HCV cure (SVR) assessment following completion of treatment, balancing potential for loss to follow up if too long and potential for false negative results if too early. More than 40% of respondents considered 12 weeks after the end of treatment would be the optimal time, followed by 4 weeks (19.4%) and 8 weeks (15.3%) (Table 5).

**Table 5:** Preferred time point for an HCV test of cure

Time point after end of treatment	Number of respondents (%)
4 weeks	19 (19.4%)
8 weeks	15 (15.3%)
12 weeks	43 (43.9%)
24 weeks	11 (11.2%)
No need for test of cure after DAA	1 (1%)
Total	98

Many findings around user preferences for future hepatitis C diagnostics − such as acceptable trade-offs between sensitivity and cost, sensitivity and ease-of-use, preferred sampling method and type of test to be used as a test of cure − are included in a separate report within this Supplement [11].

## Discussion and conclusions

We report the findings of a survey of current hepatitis testing practices and preferences for future approaches among end-users and implementers of hepatitis B and C testing services across 23 LMICs in Sub-Saharan Africa, Asia, Latin America and Eastern Europe. These findings were used alongside formal evidence reviews to inform recommendations on who to test and how to test (assays and algorithms) in the 2017 WHO Guidelines on hepatitis B & C testing [9]. The majority of the respondents were clinicians and researchers, with more than 10 years’ experience in hepatitis programmes, and were able to provide an assessment of HBV and/or HCV testing practices at a national level.

There were several key findings. Overall, the results showed that HBV and HCV testing programmes are very limited in LMICs. First, only a small proportion of hepatitis testing (and often only specific populations) is currently funded through government-supported programmes. Second, testing remains largely doctor-led and only a few countries have expanded to testing by lay or unskilled health workers. Third, although testing is recommended in several populations, particularly pregnant women, children born to HBV infected mothers, health-care workers, those with HIV, and people who inject drugs (PWID), this is not well implemented and is even much lower for HCV. Very few countries are doing population-based screening. Fourth, there is a wide variation in the serological assays used, and none are WHO pre-qualified. There is also very limited access to virological testing, which means most current programmes are unable to identify persons with active infection in need of treatment.

### Who to test

Blood donor testing was widely recommended and undertaken in accordance with WHO recommendations for universal testing [12]. Although screening for other high-risk groups (health-care workers, pregnant women and people living with HIV) is recommended in the majority of LMICs represented in the survey, this is not routinely offered and remains doctor-driven and erratically implemented. The majority of respondents considered that testing should be offered in all high-risk populations listed in the survey. The 2017 WHO Guidelines now recommend that focused testing should be offered to all adults and adolescents from populations most affected by HBV or HCV infection, i.e. those who are either part of a population with high prevalence (e.g. some mobile/migrant populations from high/intermediate endemic countries, and certain indigenous populations) or who have a history of exposure and/or high risk behaviours for HBV or HCV, independent of prevalence rate (e.g. PWID, people in prisons and other closed settings, MSM, sex workers, HIV-infected persons, partners, family members and children of HBV infected persons) [9]. There is a need for further studies to examine cost-effectiveness of different testing approaches to determine the optimal mix of targeted and generalised testing approaches in settings with different epidemiological profiles. The guidelines also recommend that in countries with intermediate (>2%) and high (≥5%) seroprevalence of HBsAg or HCV antibody, general population testing should be implemented using existing testing facilities and programmes, such as HIV and TB services [9]. Finally, the WHO testing guidelines recommended that in all settings screening of blood donors should be mandatory with linkage to care, counselling and treatment for those who test positive.

### How to test

The survey respondents identified that current testing was based on HBsAg and HCV antibody serological assays only, and there was very limited access to virological testing to enable identification of those in need of treatment. The 2017 WHO testing guidelines recommend testing with a single quality-assured test to detect HBsAg or HCV antibodies followed by testing of positive individuals for HBV DNA or HCV RNA [9]. Since only a proportion of those who are HBsAg positive will require treatment, assessment of HBV viral load through NAT is critical for timely treatment decisions and monitoring treatment response. HCV RNA testing is also important to confirm viraemic infection and need for treatment, as approximately 15 to 45% of those who are HCV antibody positive will have spontaneously cleared the virus. Moreover, HBV DNA and HCV RNA are the only markers detectable during a pre-seroconversion period and hence blood supplies not tested for viraemia represent an important source of new infections [13, 14].

No respondent reported systematic use of HCV core antigen for diagnosis of HCV viraemia in LMICs, although the Abbott Architect platform (which can be used for HCV core antigen assay) was available in six of the 23 LMICs represented in the survey responses. The HCV core antigen assay available on Abbot’s Architect platform has been reported to be clinically useful in HCV diagnosis although its sensitivity is slightly lower than that of the HCV RNA test [15, 16]. A recent systematic review of studies demonstrated a strong correlation between the levels of HCV RNA and HCV core antigen [16]. The WHO testing guidelines made a conditional recommendation to consider use of HCV core antigen testing as a possible alternative to molecular HCV RNA NAT [9].

Periodic monitoring of HCV RNA during treatment is no longer necessary for most people with highly curative, well-tolerated DAA regimens [17]. A single test of cure following completion of treatment is now considered sufficient to assess treatment outcome. Half of the survey respondents indicated a preference for 12 weeks after the end of treatment followed by 4 and 8 weeks. An HCV RNA at 12 weeks (sustained virological response, SVR12) is now recommended in the WHO Guidelines [9].

However, access to a molecular and core antigen test is hampered by high cost and insufficient laboratory structure in LMICs. Factors that lead to high rates of loss to follow-up include poor linkage to laboratory testing and treatment both of which are mostly localized in central hospitals. New confirmatory tests that can be performed at lower implementation settings, and that allow making treatment decisions within the same day, would substantially improve HBV and HCV management. Target product profiles for near-patient HCV diagnosis have recently been developed [11].

There were several key limitations to this study. The survey population was based on the listserve of the WHO Global Hepatitis Programme contacts database which comprises 306 persons from a range of clinical, industry, non-governmental organizations (NGO) and civil society background, and was conducted over a limited time period of a few weeks. As a result, our sample size was relatively small and comprised 48 respondents from 23 out of a total of 135 LMICs, and there was not comprehensive geographic coverage. Only half of respondents reported knowledge of testing practices at the national level. In addition, we did not attempt to verify the accuracy of responses regarding national testing practices and policies.

The survey data shows that in most LMICs, national viral hepatitis programmes are either non-existent or in a very early stage. Survey respondents also identified the future priorities for scale-up of hepatitis testing. These are: improved public education and awareness as well as education among health-care workers, availability of testing sites, funding for HCV testing, provision of quality-assured RDTs that meet WHO performance criteria of sensitivity and specificity, and development of national guidelines and policies. There are opportunities to expand HBV and HCV testing capacity and reduce costs through use of existent HIV and TB testing services and programmes, as well as existing resourced laboratory infrastructure, platforms and personnel [18, 19]. In addition, many laboratory-based and near-patient platforms already offer multi-analyte detection [20]. There is a particular need to expand the currently very limited access to virological testing in LMICs to identify those in need of treatment for HBV and HCV. Development and implementation of new near-patient virological tests is currently ongoing to help overcome staff and resource shortages in LMICs.

## Additional files

Additional file 1: The V&P survey questionnaire. Text file in Microsoft Word format. (DOCX 50 kb)

Additional file 2: Text file in Microsoft Word containing tables describing HCV treatment availability and testing strategies used across different testing programmes. (DOCX 23 kb)
