# Supplementary material for: Values, preferences and current hepatitis B and C testing practices in low- and middle-income countries: results of a survey of end users and implementers
Source: BMC Infect Dis. 2017 Nov 1;17(Suppl 1):702. doi: 10.1186/s12879-017-2769-y (PMC5688454; doi:10.1186/s12879-017-2769-y)
Supplement: Supplementary file 1 — The V&P survey questionnaire. Text file in Microsoft Word format. (DOCX 50 kb) [file 12879_2017_2769_MOESM1_ESM.docx]

**Additional file 1.**

**The V&P survey questionnaire.**

**PART 1: Information about you**

1. **Country:** *Please select one country for which you are most qualified to discuss current practices for HCV and HBV testing***.**

Click here to enter text.

*If the selected country is not a LMIC, PARTS 2 and 3 is automatically skipped and the respondent is sent directly to PART 4.*

1. **What is your role in relation to hepatitis testing?** *Check all that apply.*

Medical doctor/ clinical officer

Medical assistant

Primary care provider

Laboratory expert

Researcher

*In vitro* diagnostics industry personnel

Employee/Consultant of a national or international NGO

Employee of an international organization (e.g., WHO)

National programme administrator

Programme implementer

Policy maker

Activist

Other ­­­­­­­­­­­*(please specify)* Click here to enter text.

1. **How long have you been working in the field of viral hepatitis?**

Less than 1 year

1-2 years

3-5 years

5-10 years

More than 10 years

1. **What is the broadest area in which you are familiar with the HBV and HCV testing that is available?**

|  | HBV | HCV |
| --- | --- | --- |
| The health programme I work in |  |  |
| Health district |  |  |
| Province/state |  |  |
| National level |  |  |
| I am not familiar with testing |  |  |

| Other (please specify below) |  |  |
| --- | --- | --- |

Click here to enter text.

*NOTE: For the following questions in PART TWO, please provide answers about the broadest area that you indicated in question #4 above (“What is the broadest area in which you are familiar with available HBV and HCV testing?”).*

**PART 2: Information about the current viral hepatitis testing practices in your area** *(i.e. the broadest area in which you are familiar with the available HBV and HCV testing)*

1. **Is any type of HCV/HBV testing currently available?**

|  | HBV | HCV |
| --- | --- | --- |
| Yes |  |  |
| No |  |  |

1. **Which type(s) of service provider(s) offers testing?** *Check all that apply.*

|  | HBV | HCV |
| --- | --- | --- |
| Public |  |  |
| Private |  |  |
| NGO/faith-based organization *(please indicate the name(s) below)*  Click here to enter text. |  |  |

1. **What is the principal source of funding for hepatitis testing?**

|  | HBV | HCV |
| --- | --- | --- |
| Patients (i.e., self funded) |  |  |
| Government |  |  |
| Private insurance |  |  |
| Other (*please specify below)*  Click here to enter text. |  |  |

1. **Is hepatitis testing part of a programme(s) that targets any of the following populations?** *Please check all that apply and indicate which tests are used for each of the selected programmes.*

**(a) HBV testing**

RDT = rapid diagnostic test for HBV antigen (HBsAg); EIA/RIA = enzyme immunoassay/radioimmunoassay for HBsAg; DNA = nucleic acid amplification testing for HBV DNA; FS = fibrosis staging; Not sure = not sure what kind of testing is used for this

| **Specific population** | **Part of targeted programme?**  *(Check the box for “yes”)* | **Type of test** | | | |
| --- | --- | --- | --- | --- | --- |
|  | **Yes** | **RDT/EIA/RIA** | **DNA** | **FS** | **Not sure** |
| Blood donors |  |  |  |  |  |
| Health-care workers |  |  |  |  |  |
| People who inject drugs |  |  |  |  |  |
| Men who have sex with men |  |  |  |  |  |
| Migrants |  |  |  |  |  |
| Pregnant women |  |  |  |  |  |
| Children born to HCV/HBV-infected mothers |  |  |  |  |  |
| Patients who have frequent contact with the health-care system (e.g. chronically ill) |  |  |  |  |  |
| Commercial sex workers |  |  |  |  |  |
| People living with HIV |  |  |  |  |  |
| Prisoners |  |  |  |  |  |
| Population testing independent of risk factors available on a large scale |  |  |  |  |  |
| Other *(please indicate)* Click here to enter text. |  |  |  |  |  |
| Testing is not a part of any programme |  |  |  |  |  |
| I don’t know which specific populations may have targeted hepatitis testing programmes |  |  |  |  |  |

**(b) HCV testing**

RDT = rapid diagnostic test for HCV antibodies; EIA/RIA = enzyme immunoassay/radioimmunoassay for HCV antibodies; cAg = HCV core antigen test; RNA = nucleic acid amplification testing for HCV RNA; FS = fibrosis staging; Not sure = not sure what kind of testing is used for this

| **Specific population** | **Part of targeted programme?**  *(Check the box for “yes”)* | **Type of test** | | | | |
| --- | --- | --- | --- | --- | --- | --- |
|  | **Yes** | **RDT/EIA/RIA** | **RNA** | **cAg** | **FS** | **Not sure** |
| Blood donors |  |  |  |  |  |  |
| Health-care workers |  |  |  |  |  |  |
| People who inject drugs |  |  |  |  |  |  |
| Men who have sex with men |  |  |  |  |  |  |
| Migrants |  |  |  |  |  |  |
| Pregnant women |  |  |  |  |  |  |
| Children born to HCV/HBV-infected mothers |  |  |  |  |  |  |
| Patients who have frequent contact with the health-care system (e.g. chronically ill) |  |  |  |  |  |  |
| Commercial sex workers |  |  |  |  |  |  |
| People living with HIV |  |  |  |  |  |  |
| Prisoners |  |  |  |  |  |  |
| Population testing independent of risk factors available on a large scale |  |  |  |  |  |  |
| Other *(please indicate)* Click here to enter text. |  |  |  |  |  |  |
| Testing is not a part of any programme |  |  |  |  |  |  |
| I don’t know which specific populations may have targeted hepatitis testing programmes |  |  |  |  |  |  |

1. **What HBV/HCV tests are most frequently used across the selected programmes?** *Enter all brands that are commonly used. Write “Don’t know” if you do not know.*

**(a) HBV testing:**

| **Rapid diagnostic test (RDT) or enzyme immunoassay (EIA)/radioimmunoassay (RIA) for HBV antigen (HBsAg)**  (e.g. SD BIOLINE, Alere Determine, ASSURE, VIKIA, Advanced Quality, TRI-DOT, Hexagon, etc.) | Click here to enter text. |
| --- | --- |
| **HBV DNA test**  (e.g. Roche COBAS,^®^ TaqMan, Abbott RealTime, Artus,^TM^ etc.) | Click here to enter text. |

**(b) HCV testing**

| **Rapid diagnostic test (RDT) or enzyme immunoassay (EIA)/radioimmunoassay (RIA) for HCV antibodies**  (e.g. SD BIOLINE, ASSURE, OraQuick, Advanced Quality, TRI-DOT, Hexagon, Alere ImmunoComb etc.) | Click here to enter text. |
| --- | --- |
| **HCV core antigen test**  (e.g. Abbott Architect) | Click here to enter text. |
| **HCV RNA test**  (e.g. Roche COBAS,^®^ TaqMan, Abbott RealTime, Artus,^TM^ etc.) | Click here to enter text. |
| **(c) Fibrosis stage testing**  (e.g. routine blood tests, FibroTest, ActiTest, FibroScan, liver biopsy, etc.) | Click here to enter text. |

1. **Which algorithm of testing is used for the selected programmes?** *Check all that apply.*

**(a) HBV testing:**

Rapid diagnostic test (RDT)/enzyme immunoassay (EIA)/radioimmunoassay (RIA) as a stand-alone test

RDT/EIA/RIA followed by a HBV DNA test

HBV DNA test as a stand-alone test

Other (*please describe*) Click here to enter text.

Not sure

**(b) HCV testing:**

Rapid diagnostic test (RDT)/enzyme immunoassay (EIA)/radioimmunoassay (RIA) as a stand-alone test

RDT/EIA/RIA followed by HCV core antigen test

RDT/EIA/RIA followed by a HCV RNA test

HCV RNA test as a stand-alone test

HCV core antigen test as a stand-alone test

Other (*please describe*) Click here to enter text.

Not sure

1. **What healthcare professionals are involved in the hepatitis testing pathway?** *Check all that apply.*

|  | HBV | HCV |
| --- | --- | --- |
| Lab technician |  |  |
| Physician |  |  |
| Nurse |  |  |
| Midwife |  |  |
| Health-care worker |  |  |
| Other (*please specify*)  Click here to enter text. |  |  |
| Not sure |  |  |

1. **What form of counseling is provided?**

|  | HBV | HCV |
| --- | --- | --- |
| Pre-test counseling |  |  |
| Post-test counseling |  |  |
| Not sure |  |  |

**PART 3: Preferences for future hepatitis testing practices in your area**

1. **For which target population group(s) is HCV and/or HBV testing currently *not* established but you would consider it a priority to establish?** *Check all that apply.*

|  | HBV | HCV |
| --- | --- | --- |
| Blood donors |  |  |
| Health-care workers |  |  |
| People who inject drugs |  |  |
| Men who have sex with men |  |  |
| Migrants |  |  |
| Pregnant women |  |  |
| Children born to HCV- or HBV-infected mothers |  |  |
| Patients who have had frequent contact with the health-care system (e.g. chronically ill) |  |  |
| Sex workers |  |  |
| People living with HIV |  |  |
| Prisoners |  |  |
| Population testing independent of risk factors available on a large scale |  |  |
| Other *(please indicate)* Click here to enter text. |  |  |
| Other *(please indicate)* Click here to enter text. |  |  |
| Not sure |  |  |

1. **Of the following algorithms for HCV diagnosis, which one would you prefer:**
   - **Two-step testing**: a screening rapid test (for HCV antibody) followed by a laboratory-based confirmatory test (for HCV RNA or core antigen)

*or*

- - **One-step testing**: a low-cost (less than US$ 15), point-of-care HCV virological test (for HCV RNA or core antigen)

| One-step | because of *(check all that apply)* |  | Simplicity of algorithm |
| --- | --- | --- | --- |
|  |  |  | Reduced rate of loss to follow-up |
|  |  |  | High prevalence of HCV infection in the population |
|  |  |  | Low prevalence of HCV infection in the population |
|  |  |  | Other reason(s):  Click here to enter text. |

| Two-step, | because of *(check all that apply)* |  | Confirmatory laboratory-based HCV RNA assay is a gold standard for HCV diagnostics |
| --- | --- | --- | --- |
|  |  |  | High prevalence of HCV infection in the population |
|  |  |  | Low prevalence of HCV infection in the population |
|  |  |  | Other reason(s):  Click here to enter text. |

Not sure/need more information to inform a preference

1. **Under the previously proposed two-step testing algorithm, the existing confirmatory test for HCV RNA has a diagnostic sensitivity of >99% (i.e. less than 1% of test results are false negatives), while the alternative, a HCV core antigen test, costs less and could increase access to HCV diagnosis, but has a diagnostic sensitivity of 95% (i.e. 5% false negative). Considering this, which type of test would you prefer as a one-step point-of-care diagnostic test?**

|  | **Cost** | **Sensitivity** |
| --- | --- | --- |
| HCV RNA test | higher | higher |
| HCV cAg test | lower | lower |

Please explain your preference

Click here to enter text.

1. **What would you consider to be a maximum acceptable manufacturer’s price per test (i.e. price that does not include delivery or import costs) for a point-of-care solution that could be used in a one-step algorithm?**
2. **For an HCV RNA test that could be used in a one-step algorithm with 1-2% false negatives:**

More than US$ 20

US$ 11-20

US$ 1-10

Less than US$ 1

1. **For an HCV core antigen test that could be used in a one-step algorithm with 5% false negatives:**

More than US$ 20

US$ 11-20

US$ 1-10

Less than US$ 1

Such a test is not acceptable

1. **What would be *the lowest* acceptable sensitivity for a one-step HCV testing algorithm in a point-of-care setting?**

98% *(i.e. 2% false negatives)*

95% *(i.e. 5% false negatives)*

90% *(i.e. 10% false negatives)*

85% *(i.e. 15% false negatives)*

Other *(please specify)* Click here to enter text.

1. **In your opinion, how important are the following issues when establishing large-scale HCV testing in low- and middle-income countries?**

| **(a) Patient perspective** |  |  |  |  |  |
| --- | --- | --- | --- | --- | --- |
|  | **Not important** | **Moderately important** | **Important** | **Very important** | **Not sure** |
| Public education and awareness |  |  |  |  |  |
| Lack of social support and stigmatization |  |  |  |  |  |
| Loss to follow-up (people not returning for their test results) |  |  |  |  |  |
| Loss to follow-up of people not returning for final test-of-cure results after completion of therapy |  |  |  |  |  |
| **(b) Service provider perspective** | |  |  |  |  |
|  | **Not important** | **Moderately important** | **Important** | **Very important** | **Not sure** |
| Lack of knowledge among health professionals |  |  |  |  |  |
| Lack of counseling services |  |  |  |  |  |
| Service capacity:   - Lack of clinical staff to perform HCV rapid diagnostic tests - Lack of lab staff to perform laboratory-based tests |  |  |  |  |  |
| **(c) Health system perspective** | |  |  |  |  |
|  | **Not important** | **Moderately important** | **Important** | **Very important** | **Not sure** |
| Availability of diagnostic sites |  |  |  |  |  |
| Availability of on-site confirmatory testing |  |  |  |  |  |
| Funding for HCV testing |  |  |  |  |  |
| Lack of national policies and guidelines |  |  |  |  |  |
| **(d) Technology perspective** | |  |  |  |  |
|  | **Not important** | **Moderately important** | **Important** | **Very important** | **Not sure** |
| Quality-assured rapid diagnostic tests for HCV antibodies (RDTs) |  |  |  |  |  |
| Sensitivity/specificity of RDTs |  |  |  |  |  |
| Phlebotomy services |  |  |  |  |  |
| Complexity of existing diagnostic algorithm |  |  |  |  |  |
| The need for further referral for confirmatory testing |  |  |  |  |  |
| Quality-assured dried blood spot testing |  |  |  |  |  |
| Barriers to undertake testing due to:   - No instruments available - Instruments available but not operated/serviced |  |  |  |  |  |
| Access to polyvalent platforms for integrated testing of HIV, HBV and HCV |  |  |  |  |  |
| Difficulties with sample transport |  |  |  |  |  |
| Turn-around time of test results |  |  |  |  |  |
| Other, please specify:  Click here to enter text. |  |  |  |  |  |
| Other, please specify:  Click here to enter text. |  |  |  |  |  |

1. **In your opinion, how much of a barrier is the actual price (the market price that includes test cost, delivery cost and margins) for each of the following tests (you can choose the same answer for more than one test):**

|  | **actual**  **cost per test** | **not a barrier** | **minor barrier** | **moderate barrier** | **major barrier** |
| --- | --- | --- | --- | --- | --- |
| Rapid Diagnostic Test for HCV antibodies/HBV antigen (HBsAg) | US$ 1-8 |  |  |  |  |
| HCV Core antigen test | ~ US$ 30 |  |  |  |  |
| HCV RNA test | ~ US$ 50 |  |  |  |  |
| HBV DNA test | ~ US$ 50 |  |  |  |  |

1. **The viral hepatitis test performed from capillary (fingerstick) blood may result in a *decrease* in sensitivity as compared to venous blood (which requires a blood draw by a phlebotomist). However, capillary sampling is easier for point-of-care testing. Given these trade-offs, which blood specimen would you prefer for HBV/HCV diagnosis?**

Capillary

Venous

Not sure/need more information

What is your motivation for your response?

Click here to enter text.

1. **Some tests are performed on plasma (a component of whole blood) instead of whole blood. How important is it to have the plasma separation integrated into the test device?**

Very important

Important

Moderately important

Not important

Not sure/need more information

1. **In your opinion, how important is it to be able to perform viral hepatitis testing using dried blood spots (i.e. blood samples can be collected and dried on a special card in point-of-care settings and then sent to a centralized laboratory for analysis)?**

**(a) DBS testing for HBV antigen (HBsAg) and HCV antibodies**

|  | HBV | HCV |
| --- | --- | --- |
| Very important |  |  |
| Important |  |  |
| Moderately important |  |  |
| Not important |  |  |
| Not sure |  |  |

**Please explain your preferences for each:**

|  | HBV | HCV |
| --- | --- | --- |
| Ease of samples attainment |  |  |
| Ease of transport |  |  |
| Minimal need for return visits |  |  |
| Longer turnaround time |  |  |
| Lower sensitivity |  |  |
| Other (please specify)  Click here to enter text. |  |  |

**(b) DBS testing for HBV DNA and HCV RNA**

|  | HBV | HCV |
| --- | --- | --- |
| Very important |  |  |
| Important |  |  |
| Moderately important |  |  |
| Not important |  |  |
| Not sure |  |  |

**Please explain your preferences for each:**

|  | HBV | HCV |
| --- | --- | --- |
| Ease of samples attainment |  |  |
| Ease of transport |  |  |
| Minimal need for return visits |  |  |
| Longer turnaround time |  |  |
| Lower sensitivity |  |  |
| Other (please specify)  Click here to enter text. |  |  |

1. **How long should be the *maximum* acceptable interval between taking the sample and returning the HCV test result in order to maximize the potential health impact?**

30 minutes

1 hour

2 hours

Longer than 2 hours but available the same day

Results available the next day or later

Other, please specify:

Please explain your preference

Click here to enter text.

1. **Thinking about the need for viral hepatitis testing in your area and available medical personnel. In order to effectively scale up the testing to meet the need, what level of healthcare worker should be able to perform a point-of-care HBV/HCV test?** *Check all that apply*

Community health-care workers (least training)

Lab staff

Nurses

Doctors (most training)

**PART 4: HCV treatment and the test of cure**

1. **At which level(s) of the health-care system is HCV treatment available?** *Check all that apply.*

National hospital (Level IV)

Regional or referral hospital (Level III)

District hospital (Level II)

Health centre (Level I)

Rural clinic / Community centre (Level 0)

Private clinic

NGO clinic

Treatment is provided, but not sure at which health-care level(s)

No treatment provided

Not sure if treatment is provided

*If multiple levels were selected:*

**At which level of the health system do the majority of patients receive HCV treatment?**

*Choice between previously selected levels*

1. **Which of the following sites do you think are moderately or highly likely to be used for HCV treatment in the next 5 years in your area?** *Check all that apply*.

National hospital (Level IV)

Regional or referral hospital (Level III)

District hospital (Level II)

Health centre (Level I)

Rural clinic / Community centre (Level 0)

Private clinic

NGO clinic

Not sure

1. **Although having one test device for both HCV diagnosis and the test of cure is cheaper and easier to perform, it limits the settings in which they can be used. Having two different tests would allow the health system to use them in different settings (e.g. a low-cost one-step point-of-care test for HCV diagnosis and “gold standard” laboratory-based HCV RNA test as the test of cure). Please indicate which of the following would be most beneficial in your area.**

Same test: HCV RNA test in a centralized setting

Same test: HCV core antigen test in a centralized setting

Same test: HCV RNA in a decentralized setting

Same test: HCV core antigen test in a decentralized setting

Different tests: a decentralized HCV RNA / Core antigen test for diagnosis and centralized HCV RNA test for treatment response monitoring

Not sure/Need more info to express preference

**Longer timing between end of HCV treatment and test of cure could result in greater loss to follow-up. However, performing the test of cure too soon after treatment can result in false negative tests.**

**Keeping this in mind, how long do you think you could reasonably wait before performing a test of cure without increasing your rate of loss to follow-up?**

4 weeks

8 weeks

12 weeks

24 weeks

No need for the test of cure after direct-acting therapy

Not sure

Please explain your preference

Click here to enter text.

1. **Is there anything else you would like to tell us about HCV or HBV diagnostic testing in low- and middle-income countries?**

Click here to enter text.
