# Supplementary material for: Values, preferences and current hepatitis B and C testing practices in low- and middle-income countries: results of a survey of end users and implementers
Source: BMC Infect Dis. 2017 Nov 1;17(Suppl 1):702. doi: 10.1186/s12879-017-2769-y (PMC5688454; doi:10.1186/s12879-017-2769-y)
Supplement: Supplementary file 2 — Text file in Microsoft Word containing tables describing HCV treatment availability and testing strategies used across different testing programmes. (DOCX 23 kb) [file 12879_2017_2769_MOESM2_ESM.docx]

**Additional file 2.**

**Table 1.** HCV treatment availability.

| **Level of the healthcare system** | **Treatment available,**  **number of LMIC**  **(% of LMIC responded)** |
| --- | --- |
| National hospital (Level IV) | 18 (78.3%) |
| Regional or referral hospital (Level III) | 16 (69.6%) |
| District hospital (Level II) | 11 (47.8%) |
| Health centre (Level I) | 3 (13%) |
| Rural clinic / Community centre (Level 0) | 1 (4.3%) |
| Private clinic | 16 (69.6%) |
| NGO clinic | 2 (8.7%) |
| Treatment is provided, but not sure at which health-care level(s) | 1 (4.3%) |
| Not sure | 0 |
| **Total** | **23** |

**Table 2**. Testing strategies used across different programmes.

| **Testing strategy** | **Number of LMIC**  **(% of LMIC responded)** | | |
| --- | --- | --- | --- |
|  | | |  |
| **HBV testing** | | |  |
| RDT/EIA/RIA as a stand-alone test | | 12 (60%) | |
| RDT/EIA/RIA followed by a HBV DNA test | | 13 (65%) | |
| HBV DNA test as a stand-alone test | | 0 | |
| Not sure | | 1 (5%) | |
| **Total** | | **20** | |
|  | |  | |
| **HCV testing** | |  | |
| RDT/EIA/RIA as a stand-alone test | | 11 (50%) | |
| RDT/EIA/RIA followed by a HCV RNA test | | 14 (63.6%) | |
| HCV RNA test as a stand-alone test | | 2 (9.1%) | |
| RDT/EIA/RIA followed by HCV cAg test | | 3 (13.6%) | |
| HCV cAg test as a stand-alone test | | 0 | |
| Not sure | | 1 (4.5%) | |
| **Total** | | **22** | |
